# The Small Protein RmpD Drives Hypermucoviscosity in Klebsiella pneumoniae

**DOI:** 10.1128/mBio.01750-20

**Published:** 2020-09-22

**Authors:** Kimberly A. Walker, Logan P. Treat, Victoria E. Sepúlveda, Virginia L. Miller

**Affiliations:** a Department of Microbiology and Immunology, University of North Carolina School of Medicine, Chapel Hill, North Carolina, USA; b Department of Genetics, University of North Carolina School of Medicine, Chapel Hill, North Carolina, USA; New York University School of Medicine

**Keywords:** RmpA, RmpC, hypervirulent, HMV, capsule, hypermucoviscous

## Abstract

Klebsiella pneumoniae has a remarkable ability to cause a wide range of human diseases. It is divided into two broad classes: classical strains that are a notable problem in health care settings due to multidrug resistance, and hypervirulent (hv) strains that are historically drug sensitive but able to establish disease in immunocompetent hosts. Alarmingly, there has been an increased frequency of clinical isolates that have both drug resistance and hv-associated genes. One such gene, *rmpA*, encodes a transcriptional regulator required for maximal capsule (*cps*) gene expression and confers hypermucoviscosity (HMV). This link has resulted in the assumption that HMV is caused by elevated capsule production. However, we recently reported a new *cps* regulator, RmpC, and Δ*rmpC* mutants have reduced *cps* expression but retain HMV, suggesting that capsule production and HMV may be separable traits. Here, we report the identification of a small protein, RmpD, that is essential for HMV but does not impact capsule. RmpD is 58 residues with a putative N-terminal transmembrane domain and highly positively charged C-terminal half, and it is conserved among other hv K. pneumoniae strains. Expression of *rmpD* in *trans* complements both Δ*rmpD* and Δ*rmpA* mutants for HMV, suggesting that RmpD is the key driver of this phenotype. The *rmpD* gene is located between *rmpA* and *rmpC*, within an operon regulated by RmpA. These data, combined with our previous work, suggest a model in which the RmpA-associated phenotypes are largely due to RmpA activating the expression of *rmpD* to produce HMV and *rmpC* to stimulate *cps* expression.

## Abstract (precis)

Capsule is a critical virulence factor in Klebsiella pneumoniae, in both antibiotic-resistant classical strains and hypervirulent strains. Hypervirulent strains usually have a hypermucoviscosity (HMV) phenotype that contributes to their heightened virulence capacity, but the production of HMV is not understood. The transcriptional regulator RmpA is required for HMV and also activates capsule gene expression, leading to the assumption that HMV is caused by hyperproduction of capsule. We have identified a new gene (*rmpD*) required for HMV but not for capsule production. This distinction between HMV and capsule production will promote a better understanding of the mechanisms of hypervirulence, which is in great need given the alarming increase in clinical isolates with both drug resistance and hypervirulence traits.

## INTRODUCTION

Klebsiella pneumoniae has classically been considered an opportunistic pathogen associated with infection of immunocompromised patients in nosocomial settings (1, 2). Most infections are caused by classical K. pneumoniae (cKp) strains and present as pneumonias or urinary tract infections, sometimes leading to bacteremia and septic shock. The widespread occurrence of extended-spectrum β-lactam-resistant and carbapenem-resistant strains has led both the CDC and WHO to categorize K. pneumoniae at the highest level of concern for antibiotic resistance threats (3, 4). In addition, colistin- and tigecycline-resistant strains of K. pneumoniae have been isolated, severely limiting treatment options (5). The deadly case of a pan-resistant cKp strain (resistant to 26 antibiotics) underscores the immense challenge of treating *Klebsiella* infections (6).

In contrast to cKp, hypervirulent K. pneumoniae (hvKp) is community acquired by immunocompetent individuals (7). The pathology of hvKp is more severe than that typical of cKp and can include pyogenic liver abscesses, necrotizing fasciitis, meningitis, and endophthalmitis (8, 9). Of particular concern is the emergence of strains with both hypervirulent (hv)-associated genes or traits and the multidrug resistance that is characteristic of cKp (10). Antibiotic resistance genes are often carried on plasmids (11, 12), and many of the genes corresponding to hypervirulence are carried on large virulence plasmids or mobile genetic elements incorporated in the chromosome (13–15). That these genetic entities can be horizontally transferred suggests there is an increased risk of strains acquiring both hypervirulence and multidrug resistance (16, 17). Alarmingly, there have been recent reports of extensively resistant hypervirulent K. pneumoniae (18, 19), and multiple strains where both hv-associated genes and antimicrobial resistance genes were present on the same mobile vector (20–23). These reports of convergence of hypervirulence and antimicrobial resistance in the same strain have heightened the need to better understand how hypervirulence genes interface with a strain’s genetic background to confer hypervirulent phenotypes. This is particularly important given the extensive diversity of genetic content between K. pneumoniae strains.

*Klebsiella* virulence is largely attributable to lipopolysaccharide (LPS), pili, a polysaccharide capsule, and siderophores, and these are present in virtually all pathogenic strains (2). Features specifically linked to hypervirulence include additional siderophores (2, 24), tellurite resistance (25, 26), and hypermucoviscosity (HMV) (2, 27, 28). Capsule is also linked to hypervirulence, as the majority of hvKp strains have type K1 or K2 (24), although hv-associated traits have been found in strains with other capsule types (29). Compared to cKp, hvKp produces a thick “hypercapsule” that is thought to contribute to the HMV phenotype.

RmpA is a LuxR-like transcriptional regulator frequently encoded on virulence plasmids or on integrative chromosomal elements (ICE*Kp*) and was initially discovered as a regulator of HMV (14, 27). While the strong correlation between the presence of *rmpA* and hypervirulence has made it a key biomarker for hvKp (24, 30), we still know very little about how *rmpA* contributes to HMV and hypervirulence. Previous studies established that loss of *rmpA* decreases capsule (*cps*) gene expression and reduces HMV in commonly used hvKp strains (31, 32). We recently confirmed these *rmpA*-dependent phenotypes in the hvKp strain KPPR1S (28). We also described another regulator of capsule gene expression, RmpC, which is encoded downstream of *rmpA*; *rmpA* and *rmpC* are cotranscribed from the same promoter that is positively regulated by RmpA (28). Like *rmpA* mutants, the *rmpC* mutant showed reduced *cps* gene expression but, unlike *rmpA* mutants, retained HMV. We further showed that overexpression of *rmpA* in the wild type (WT) or the Δ*rmpA* and Δ*rmpC* mutants increased HMV. However, overexpression of *rmpA* did not restore *cps* expression in the Δ*rmpC* strain, and overexpression of *rmpC* elevated *cps* expression even in the Δ*rmpA* strain (28). These data suggest that (i) RmpA is an important determinant for HMV but RmpC is not, (ii) reduced *cps* expression in the Δ*rmpA* strain is likely a consequence of reduced *rmpC* expression rather than direct regulation by RmpA, and (iii) high levels of *cps* expression are not necessary to confer HMV. The latter conclusion stems from the fact that the Δ*rmpC* mutant has reduced *cps* expression and capsule production (by uronic acid assay) but retains the HMV phenotype and that exogenous expression of *rmpA* in the Δ*rmpC* strain results in elevated HMV without restoring *cps* expression (28). Importantly, this was the first clear evidence of a separation between capsule expression and HMV and suggests that HMV is not simply a consequence of elevated capsule production.

Here, we report the discovery of a small protein, RmpD, encoded between *rmpA* and *rmpC* that is essential for HMV. The Δ*rmpD* mutant is non-HMV, has no change in *cps* expression, and produces the same amount of uronic acid (capsule) as the wild-type parental strain. This provides corroborating evidence that HMV and capsule production result from distinct processes. Expression of *rmpD* is sufficient to confer HMV to a Δ*rmpA* mutant. It is transcribed by the promoter upstream of *rmpA* and therefore is also regulated by RmpA. Thus, it appears that the loss of HMV and *cps* expression observed in *rmpA* mutants is due to reduced transcription of *rmpD* and *rmpC*, respectively, and that the contribution of RmpA to these phenotypes is as an activator of this operon.

## RESULTS

### RmpD is required for hypermucoviscosity.

**FIG 1:**
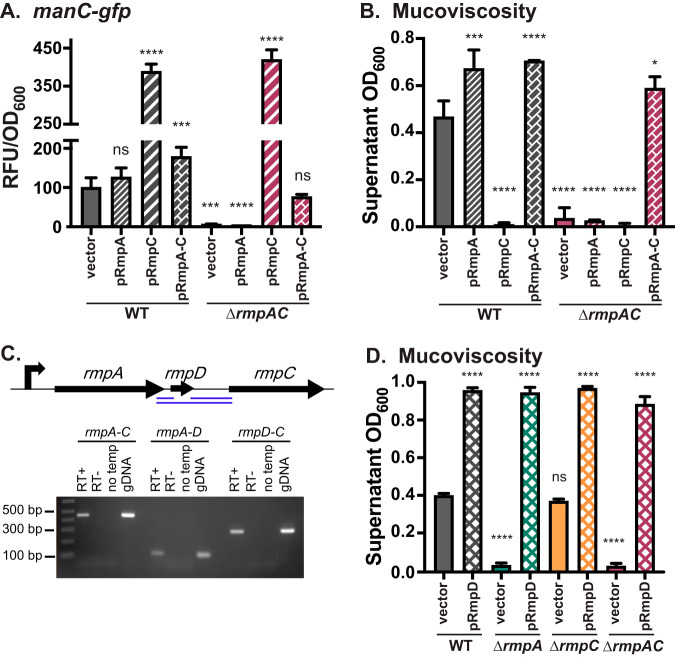
RmpD is required for HMV. Following transformation of the Δ*rmpAC* mutant with pRmpA, pRmpC, or pRmpA-C, *manC* expression (A) and mucoviscosity (B) were assayed as described in Materials and Methods. (C) Schematic of the *rmp* locus (top) and operon structure (bottom). Reverse transcription-PCR (RT-PCR) was performed using primers positioned near the 5′ or 3′ end of the three genes to amplify DNA fragments (blue lines) spanning the intergenic regions. Products were separated on a 1% agarose gel. PCR templates: RT+, products from cDNA synthesis reaction; RT−, cDNA synthesis without reverse transcriptase; no temp, PCR with no cDNA added; gDNA, genomic DNA (positive control). (D) Effect on mucoviscosity of *trans* expression of pRmpD in WT, Δ*rmpA*, Δ*rmpC*, and Δ*rmpAC* strains. One-way analysis of variance (ANOVA) with Dunnett’s multiple-comparison test was performed using WT with vector as the reference. ns, not significant; ****, *P* < 0.0001; ***, *P* < 0.001; *, *P* ≤ 0.05. Data were obtained after a 6-h induction of plasmid-borne *rmp* genes.

Having observed that hypermucoviscosity (HMV) is not necessarily a consequence of elevated capsule expression from examining the individual Δ*rmpA* and Δ*rmpC* strains with complementation plasmids (28), we took one further step by similarly testing a strain that lacked the region harboring both *rmpA* and *rmpC* (strain Δ*rmpAC*). We predicted that introduction of pRmpA would restore HMV and that pRmpC would restore *cps* expression. Because the string test for HMV is qualitative, HMV was assessed by measuring the optical density at 600 nm (OD_600_) of culture supernatants following low-speed centrifugation, an established assay for HMV (31–34); all string test-negative strains pellet tightly, while string test-positive strains remain turbid. Expression of capsule genes was monitored using a promoter-green fluorescent protein (GFP) reporter fused to *manC* (*manC* encodes an enzyme that makes one of the K2 sugar precursors and is located in the *cps* locus). The *manC* promoter is the third of three characterized promoters in the capsule locus. The *galF* promoter is regulated by RmpA and RmpC in a similar manner as *manC*, and the *wzi* promoter is not regulated by these proteins (28). While pRmpC resulted in elevated *manC* levels in the Δ*rmpAC* mutant as expected, pRmpA failed to restore HMV in this mutant (Fig. 1A and B). However, introduction of a plasmid containing the entire region that was deleted resulted in elevated HMV, suggesting an element contained within the intergenic space was necessary for HMV. In examining this region, the open reading frame (ORF) prediction function in Geneious R11 identified a small ORF between *rmpA* and *rmpC* (Fig. 1C). We cloned this predicted ORF with its putative ribosome binding site (RBS) into pMWO-078, transformed it into KPPR1S, Δ*rmpA*, Δ*rmpC*, and Δ*rmpAC* strains, and assayed for HMV (Fig. 1D). Introduction of this plasmid (pRmpD) resulted in a hyper-HMV phenotype in all strains, including the Δ*rmpAC* strain. Thus, this gene is required for HMV and was named *rmpD*. Transcriptional readthrough of the intergenic regions between *rmpA*-*rmpD* and *rmpD*-*rmpC* indicates *rmpD* is within the *rmp* operon (Fig. 1C) that is autoregulated by RmpA (28).

**FIG 2:**
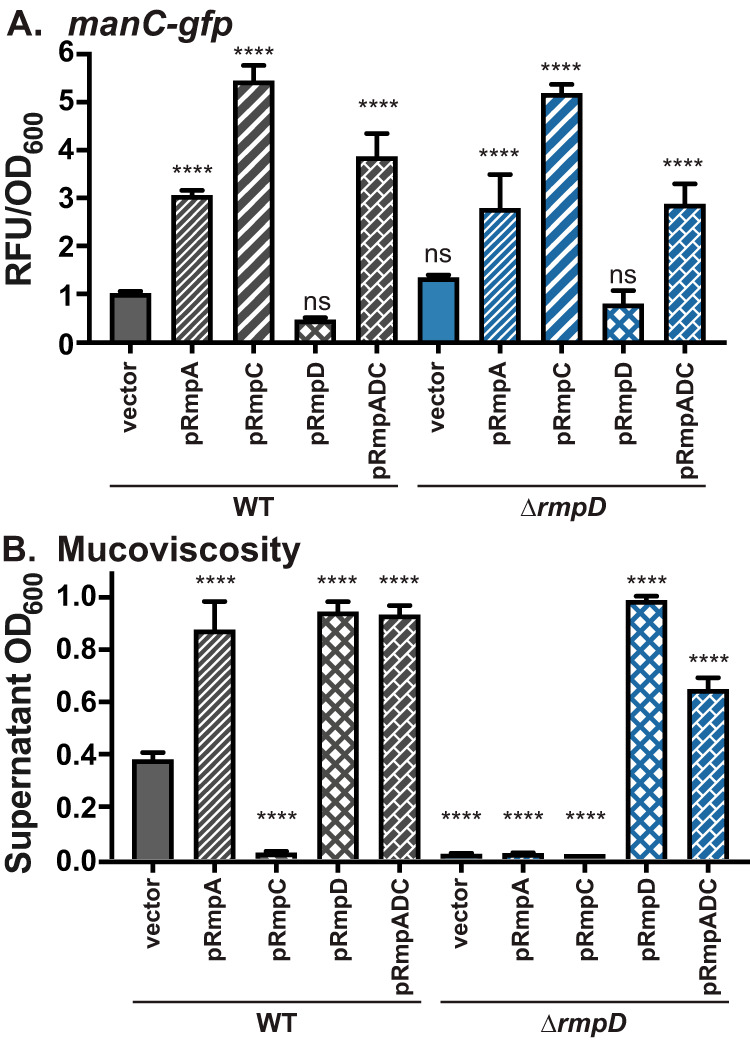
Analysis of Δ*rmpD* strain indicates RmpD but not RmpA is required for HMV. *manC-gfp* expression (A) and mucoviscosity (B) were measured in WT and Δ*rmpD* strains with the indicated plasmids as described in Materials and Methods. Data were obtained after a 6-h induction of plasmid-borne *rmp* genes. One-way ANOVA with Dunnett’s posttest was used to determine significance using WT with vector as the reference. ns, not significant; ****, *P* < 0.0001.

To further analyze the role of *rmpD*, we constructed a strain lacking *rmpD* (Δ*rmpD*) and examined *manC* expression and HMV in this mutant. The Δ*rmpD* mutant had wild-type levels of *manC* expression and was non-HMV (Fig. 2). The other known promoters in the capsule locus (*galF* and *wzi*) showed no expression defects in the Δ*rmpD* mutant (see Fig. S1 in the supplemental material). Supporting the notion that it is *rmpD* and not *rmpA* that is necessary for HMV, pRmpA was unable to restore HMV in the Δ*rmpD* mutant. Introduction of pRmpC into the Δ*rmpD* strain resulted in the same high levels of *manC* expression observed in other strains but did not restore HMV. Complementation of Δ*rmpD* with pRmpADC (formerly pRmpA-C) also resulted in an elevated level of HMV. The cultures in which *rmpD* is overexpressed become extremely viscous and have the consistency of a thick syrup (see Fig. S2) but have no change in transcription of *manC* (Fig. 2). Collectively, these data suggest that *rmpD* is directly necessary for HMV. Given that RmpA regulates the promoter driving expression of *rmpADC* (28), the well-established role of RmpA as a requisite factor for the HMV phenotype is likely due to its function as a transcriptional activator of *rmpD* expression.

10.1128/mBio.01750-20.1 FIG S1 Expression of other known capsule promoters is not deficient in the Δ*rmpD* strain. Promoter regions upstream of *galF* (A) and *wzi* (B) were cloned into pPROBE, and fluorescence was measured as an indicator of promoter activity as described in Materials and Methods. Values were normalized to that of the WT for each promoter. The increase in expression of *galF* in the Δ*rmpD* mutant is significantly higher than in the WT (*P* < 0.0001). Download FIG S1, PDF file, 0.08 MB. Copyright © 2020 Walker et al. 2020 Walker et al. This content is distributed under the terms of the Creative Commons Attribution 4.0 International license.

10.1128/mBio.01750-20.2 FIG S2 K. pneumoniae overexpressing *rmpD* is thick and syrupy. Cultures were grown with induction by aTc as described in Materials and Methods; 100 μl of culture was dropped into 900 μl 1× phosphate-buffered saline (PBS) in a spectrophotometer cuvette. Download FIG S2, PDF file, 2.5 MB. Copyright © 2020 Walker et al. 2020 Walker et al. This content is distributed under the terms of the Creative Commons Attribution 4.0 International license.

### *rmpD* is conserved among hvKp strains and encodes a protein.

**FIG 3:**
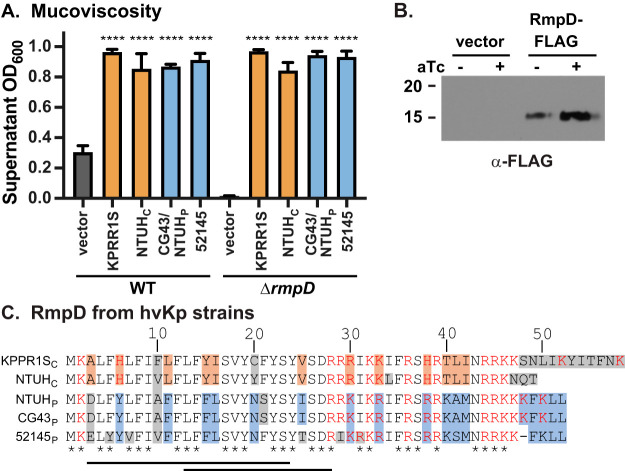
*rmpD* encodes a protein that is conserved among hypervirulent K. pneumoniae. (A) *rmpD* genes from several hvKp strains were identified using Geneious Prime, cloned, and expressed in either WT or Δ*rmpD* strains, which were assayed for mucoviscosity. One-way ANOVA with Tukey’s posttest was used to determine significance using the WT or the Δ*rmpD* mutant with vector as the reference. ****, *P* < 0.0001. (B) Western blot analysis of whole-cell extracts from WT carrying pRmpD-2×FLAG probed with α-FLAG antibody. (C) RmpD from known hvKp strains. c, chromosomal copy; p, plasmid copy; orange and blue boxes, residues conserved in chromosomal and plasmid copies, respectively; gray boxes, nonconserved residues; *, fully conserved residues; red residues, positively charged side chains, Black lines indicate predicted transmembrane domain regions: residues 3 to 23 predicted by Geneious Prime and 13 to 28 predicted by Phyre2. Accession numbers for these sequences are in the Text S1 in the supplemental material.

Examination of other hvKp strains used frequently for experimentation revealed that *rmpD* is present in these strains (see Fig. S3A). The published sequences of two strains, KPPR1S and NTUH-K2044, have an additional ORF annotated in the *rmpA-C* intergenic region in the opposite orientation relative to that of *rmpA*. Given the operon structure, it seemed unlikely this ORF would play a role in HMV, and indeed, it does not (Fig. S3B). To determine if these *rmpD* orthologs are functionally similar, we cloned the *rmpD* ORF and putative RBS from these strains and tested them in the WT and Δ*rmpD* strains of KPPR1S. Each gene retained the ability to confer hyper-HMV in both WT and Δ*rmpD* strains (Fig. 3A), suggesting that the role of *rmpD* in HMV is conserved among various K. pneumoniae isolates.

10.1128/mBio.01750-20.3 FIG S3 Organization of the *rmp* locus in K. pneumoniae. (A) Schematic of *rmp* loci from several hvKp strains. Black genes, annotated ORFs in NCBI; gray genes, annotated ORFs in Geneious Prime 2019 software. Accession numbers for these genomes are in Text S1. (B) The ORF encoded on the opposite strand (VK055_5098) with putative RBS was cloned into pMWO-078 (pORF5098), transformed into the indicated strains, and tested for effects on mucoviscosity. It had no impact in any strain, indicating that this ORF does not contribute to the HMV phenotype. Download FIG S3, PDF file, 0.09 MB. Copyright © 2020 Walker et al. 2020 Walker et al. This content is distributed under the terms of the Creative Commons Attribution 4.0 International license.

10.1128/mBio.01750-20.7 TEXT S1 Supplemental methods. Text file containing additional detail for Methods and Materials. Download Text S1, DOCX file, 0.1 MB. Copyright © 2020 Walker et al. 2020 Walker et al. This content is distributed under the terms of the Creative Commons Attribution 4.0 International license.

Although there is a predicted ORF of 58 amino acids in the DNA sequence cloned in pRmpD (KPPR1S), there remained the possibility that this region encoded a regulatory RNA. To distinguish between these possibilities, we constructed a plasmid with an *rmpD-*2×FLAG fusion and were able to detect a FLAG-tagged protein close to the predicted size (Fig. 3B), indicating that *rmpD* encodes a protein and not a regulatory RNA. RmpD contains a putative transmembrane domain near the N terminus, and the C terminus has numerous positively charged amino acids; these features are conserved in RmpD from other hvKp strains (Fig. 3C). The sequence for RmpD was analyzed using Phyre2 (35). The secondary structure is predicted to be all α-helical and oriented with the C-terminal region in the cytoplasm; no reliable tertiary structure was predicted.

### Impact of *rmpD* in capsule mutants.

**FIG 4:**
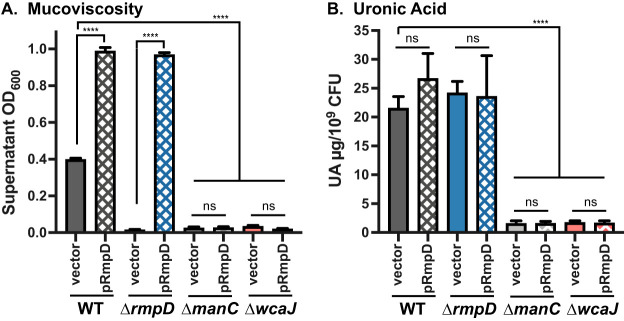
No strong correlation between capsule levels and HMV. Mucoviscosity assay (A) and uronic acid assay (B) of WT, Δ*rmpD*, Δ*manC*, and Δ*wcaJ* strains with vector (pMWO-078) or pRmpD. Data were obtained after a 6-h induction of plasmid-borne *rmp* genes as described in Materials and Methods. One-way ANOVA with Tukey’s posttest was used to determine significance to obtain all pairwise comparisons. ns, not significant; ****, *P* < 0.0001.

**FIG 5:**
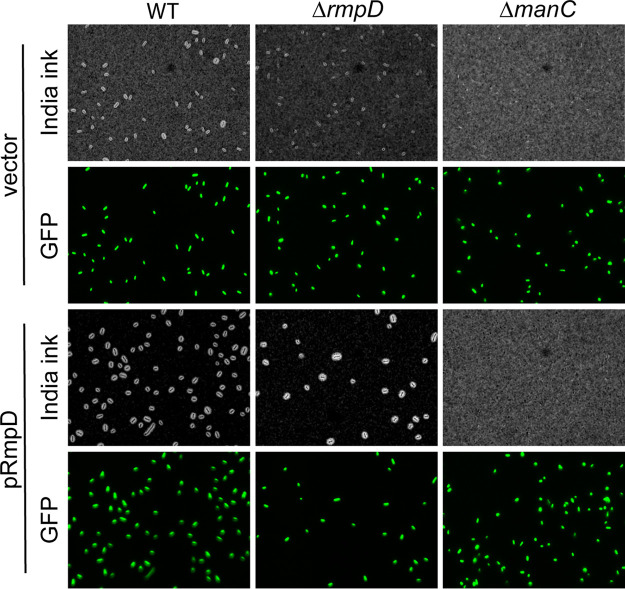
The *rmpD* mutant is encapsulated. Bacteria expressing *gfp* were stained with India ink and imaged at ×1,000 magnification. (India ink) Exopolysaccharide is visualized by a clearing zone (ink exclusion) around the bacteria. (GFP) Fluorescence images indicating the presence of the bacteria. Background shading varies due to uneven liquid distribution under the coverslip.

Contributing to assumptions in the field that HMV is derived from capsule, mutants in hvKp strains that produce no or reduced levels of capsule have also typically been reported as non-HMV (31, 32, 34, 36). To further probe the distinction between capsule production and HMV, we transformed two capsule mutants (Δ*manC* and Δ*wcaJ*) with pRmpD to determine if these strains could become hyper-HMV. *manC* encodes a GDP-mannose pyrophosphorylase that produces UDP-mannose, one of the sugar precursors of K2 capsule, and *wcaJ* encodes the initiating glycosyltransferase (undecaprenyl phosphotransferase) involved in building the four-sugar K2 subunit. Both capsule mutants, with or without pRmpD, fully sedimented following low-speed centrifugation (Fig. 4A), suggesting that the HMV phenotype requires some capsule biosynthetic enzymes and may require capsule production. We therefore examined capsule production in the Δ*rmpD* strain using the uronic acid (UA) assay. There was no decrease in UA levels in the Δ*rmpD* strain compared to that in the WT, and addition of pRmpD did not lead to increased UA (Fig. 4B). Collectively, these data imply that production of capsule is not impacted by RmpD but that at least some components of capsule must be present in order to become HMV. The negative stain, India ink, can be used to visualize capsule as zones of exclusion surrounding the bacterial cells. We stained WT, Δ*rmpD*, and Δ*manC* strains with and without pRmpD to examine the zones in these samples; all strains also expressed *gfp* to verify presence of the bacteria. WT bacteria showed exclusion zones that varied somewhat in size, whereas the *rmpD*-deficient bacteria had thinner uniform exclusion zones (Fig. 5). When *rmpD* was overexpressed in either strain, the bacteria had uniformly large exclusion zones. As predicted, no exclusion zones were observed from staining of Δ*manC* bacteria, although the field had ample bacteria present. Given that there was no difference in the amount of UA between the WT and Δ*rmpD* strains, these data suggest that the material forming the abundant exclusion zones is different from a typical UA-containing capsule and that the HMV material also excludes the stain.

**FIG 6:**
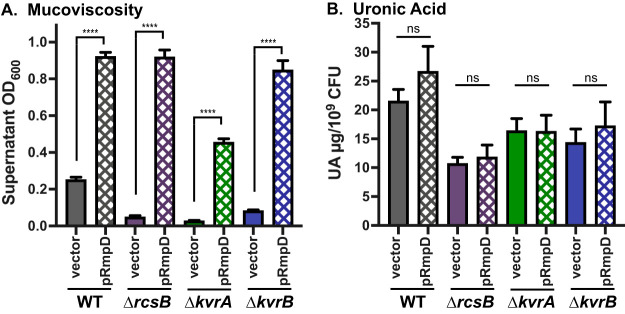
Low capsule levels are sufficient for hyper-HMV. Mucoviscosity assay (A) and uronic acid assay (B) of WT and regulatory mutants (Δ*rcsB*, Δ*kvrA*, and Δ*kvrB*) with vector (pMWO-078) or pRmpD. Data were obtained after a 6-h induction of plasmid-borne *rmpD* as described in Materials and Methods. One-way ANOVA with Tukey’s posttest was used to determine significance to obtain all pairwise comparisons. ns, not significant; ****, *P* < 0.0001.

There are several known regulators of capsule gene expression, of which, our lab has identified three and studied five (28, 36). These mutants (Δ*rmpA*, Δ*rmpC*, Δ*kvrA*, Δ*kvrB*, and Δ*rcsB*) all have reduced UA levels and capsule expression, and all but Δ*rmpC* are non-HMV. To further probe the factors necessary for HMV, we transformed the Δ*kvrA*, Δ*kvrB*, and Δ*rcsB* strains with pRmpD and assessed HMV and capsule phenotypes. When the respective deleted gene was complemented in *trans* with pKvrA, pKvrB, or pRcsB, each mutant had WT-like HMV, elevated UA levels, and elevated *manC* and *galF* expression (see Fig. S4). With pRmpD, the Δ*kvrB* and Δ*rcsB* strains became hyper-HMV similarly to the WT strain, and an intermediate level of HMV was observed for the Δ*kvrA* strain (Fig. 6A). Consistent with the results presented above, pRmpD did not restore UA production in these mutants (Fig. 6B), further indicating that strains with low capsule expression and UA production are still capable of becoming HMV.

10.1128/mBio.01750-20.4 FIG S4 Plasmids pKvrA, pKvrB, and pRcsB functionally complement their respective mutants. Mucoviscosity (A), uronic acid (B), and *manC* (C) and *galF* (D) expression were measured as described in Materials and Methods except expression data are not normalized to WT. Download FIG S4, PDF file, 0.1 MB. Copyright © 2020 Walker et al. 2020 Walker et al. This content is distributed under the terms of the Creative Commons Attribution 4.0 International license.

KPPR1S produces the enterobacterial common antigen (ECA) and LPS. A recent report showed that mutants in LPS and ECA pathways had reduced HMV (determined by sedimentation) (34, 37). To more clearly ascertain if either of these exopolysaccharides contributed to HMV, we tested a strain with a disruption in *wecA*, which encodes an undecaprenyl phosphotransferase; this mutant produces normal levels of UA but lacks both LPS and ECA and is attenuated in the pneumonia model (38). The *wecA* mutant showed WT-like HMV and became hyper-HMV when *rmpD* was overexpressed (see Fig. S5). Thus, LPS and ECA are not likely to be directly required for HMV. However, this does not rule out the possibility that other mutations in these pathways could have pleiotropic effects leading to reduced HMV. Additionally, it is possible that some enzymes required for LPS or ECA could contribute to HMV independently of their role in LPS or ECA.

10.1128/mBio.01750-20.5 FIG S5 LPS and ECA are not necessary for HMV. Uronic acid (A) and mucoviscosity (B) were measured as for Fig. 3. These data were collected preliminarily, using OD to normalize the UA concentrations. We typically normalize to CFU, but decided not to add that until we had an idea of the results. Due to COVID-19, our lab closed before we could repeat with CFU data included. The above-described assays were performed in triplicates, with three biological replicates in each assay, and so we are confident in the interpretations of these data described in Results and Discussion and did not think that statistics were needed to strengthen the analysis. Download FIG S5, PDF file, 0.08 MB. Copyright © 2020 Walker et al. 2020 Walker et al. This content is distributed under the terms of the Creative Commons Attribution 4.0 International license.

### *rmpD* contributes to immune evasion and virulence.

**FIG 7:**
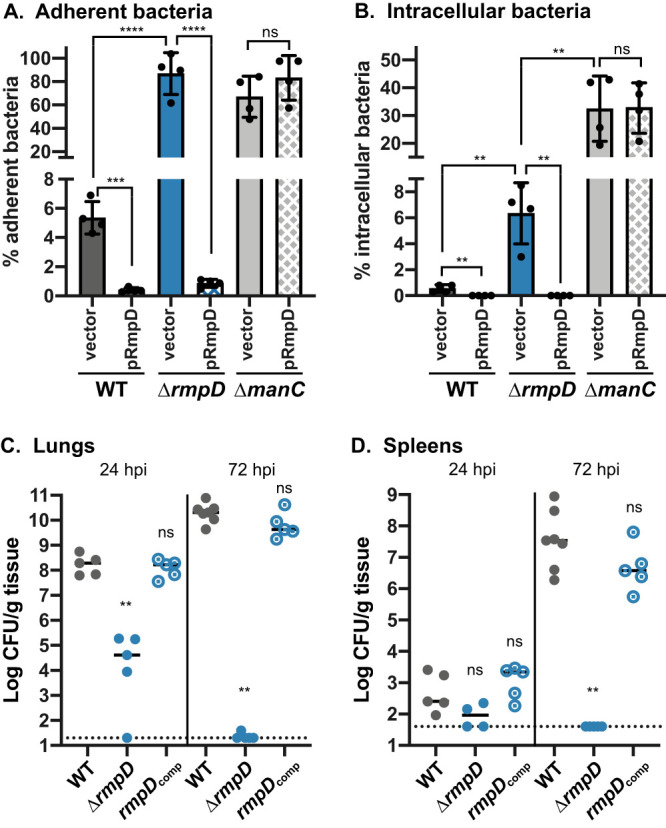
*rmpD* contributes to immune evasion and virulence. Adherence (A) and uptake (B) of bacteria were determined as described in Materials and Methods, using cytochalasin D to prevent phagocytosis (A) and gentamicin to kill extracellular bacteria (B). WT, Δ*rmpD*, and Δ*manC* strains carrying either the vector (pMWO-078) or pRmpD were tested. Two-tailed Student’s *t* test was used to determine significance. ns, not significant; ***, *P* = 0.0001; ****, *P* < 0.0001. C57BL/6J mice were inoculated with 2 × 10^4^ CFU of the indicated strains; lungs (C) and spleens (D) were harvested at 24 and 72 hpi for bacterial enumeration. Each circle represents one mouse, solid lines indicate median values, and dotted lines represent the limit of detection. Mann-Whitney test was applied to determine significance. ns, not significant; **, *P* < 0.05.

One of the virulence phenotypes associated with capsule is the blocking of adherence and phagocytosis (39). To determine if HMV specifically contributed to these processes, we performed adherence and internalization assays with the macrophage-like J774A.1 cell line. The WT strain showed approximately 5% adherence, and the Δ*manC* mutant showed nearly 70% adherence (Fig. 7A). The Δ*rmpD* strain behaved like the acapsular *manC* mutant, with ∼85% adherence. The WT or Δ*rmpD* strains with pRmpD were virtually nonadherent, with less than 1% of the bacteria attached. This reduction was not observed in the Δ*manC* mutant with pRmpD, most likely because it remains non-HMV. Because the Δ*rmpD* strain still produced capsule at the WT level and the attachment phenotype was the same as a capsule mutant, it appears that the HMV phenotype, rather than capsule *per se*, is the main factor blocking attachment to host cells. A similar trend was observed for internalization of these strains (Fig. 7B). Less than 1% of WT cells were intracellular, and approximately 35% of the Δ*manC* bacteria (with and without pRmpD) were intracellular. No bacteria were recovered from WT or Δ*rmpD* with pRmpD. Only approximately 6% of the Δ*rmpD* bacteria were internalized; this is significantly more than WT but also significantly less than the Δ*manC* mutant. These results suggest that (i) HMV blocks both adherence and internalization and (ii) capsule-positive strains are still somewhat protected against phagocytosis, even if non-HMV. Collectively, HMV appears to provide the primary barrier to adherence, while capsule and HMV each contribute to prevention of phagocytosis by macrophages.

Based on the results of the *in vitro* virulence-associated assays, we next tested the Δ*rmpD* mutant in our mouse pneumonia model and found it to be essential for virulence *in vivo*. At 24 h postinoculation (hpi), the Δ*rmpD* mutant was recovered from the lungs at levels approximately 4 logs lower than that of the WT and was essentially cleared by 72 hpi (Fig. 7C). In the spleens, the Δ*rmpD* mutant was recovered at lower numbers than the WT at 24 hpi, but WT levels were often low at this time point (Fig. 7D). However, by 72 hpi, the WT colonized the spleens to ∼10^8^ CFU/g and the *rmpD* mutant was not recovered. A strain with the *rmpD* gene restored at the native site (*rmpD*_comp_) behaved like the WT, indicating that the defects observed from Δ*rmpD* were indeed due to loss of *rmpD*.

## DISCUSSION

Hypermucoviscosity (HMV) is a phenotype possessed by a subset of K. pneumoniae strains and is one of the phenotypes associated with hypervirulent strains (2). RmpA has been established as an essential factor for HMV, and *rmpA* mutants also show reduced capsule gene expression (28, 31, 32). Thus, it has long been assumed that the HMV phenotype was a consequence of abundant capsule production in excess of that observed in classical strains. This arose despite statements in some early studies that HMV did not appear to be linked to capsule production (27, 40). However, fluorescein isothiocyanate (FITC) staining of an hv K. pneumoniae strain incubated with K2 antisera suggested the extracapsular substance associated with HMV contained capsular material (41). Although it has been 30 years since the discovery of RmpA, no direct regulation by RmpA of *cps* expression (or that of other genes) has been demonstrated. In our investigations into the contributions of RmpA to hypervirulence, we confirmed its role in HMV and *cps* expression but also ascertained that the mechanisms contributing to these phenotypes are much more complex than had been presumed (28). We identified a downstream gene encoding RmpC, a putative transcriptional regulator that modulates *cps* expression, and found that *rmpA* and *rmpC* are in an operon that is autoregulated by RmpA. RmpA and RmpC have distinct functions in that the Δ*rmpA* mutant is non-HMV but the Δ*rmpC* mutant retains HMV. Both mutants have similar reductions in *cps* expression; however, overexpression of *rmpC* complements *cps* expression even in strains lacking *rmpA*. While RmpC has also not been demonstrated to directly regulate *cps* promoters, these data indicated that RmpA was not likely to be a direct regulator of the *cps* gene. We thus concluded that RmpA controlled HMV while RmpC controlled *cps* expression in work that provided the first clear evidence separating the phenotypes of HMV and capsule levels.

In evaluating *cps* expression and HMV in what we thought was a double Δ*rmpA-rmpC* mutant, it became clear that the story was not as simple as suggested by the analysis of individual *rmpA* and *rmpC* mutants. Namely, pRmpA did not restore HMV to this Δ*rmpADC* mutant, but a plasmid containing the entire deleted region (pRmpADC) did restore HMV. In the present study, we report the initial characterization of RmpD, a small protein encoded in the region between *rmpA* and *rmpC* and within the *rmp* operon. The data presented here suggest that RmpD is the key factor driving the HMV phenotype. Collectively, our data support a model in which the role played by RmpA in the HMV and *cps* expression phenotypes is to activate expression of *rmpD* and *rmpC*. This is evidenced by (i) the restoration of HMV in the Δ*rmpA* and Δ*rmpADC* strains with pRmpD, and restoration of *cps* expression in the Δ*rmpA* and Δ*rmpADC* strains with pRmpC, and (ii) the inability of pRmpA to restore HMV in the Δ*rmpD* strain or *cps* expression in the Δ*rmpC* strain. Given that several RmpD orthologs were able to complement HMV in the Δ*rmpD* strain and that *rmpD* is present in strains that also have *rmpA* and *rmpC*, we speculate that RmpD is part of a conserved mechanism conferring HMV to K. pneumoniae. Little information can be gleaned about how RmpD acts from sequence and structural analyses. It is predicted to be α-helical and anchored in the inner membrane by an N-terminal transmembrane domain with the C terminus positioned in the cytoplasm. As the C-terminal region of all RmpD orthologs contains a number of positively charged residues, we speculate that these charges allow for specific protein-protein interactions that mediate the HMV phenotype.

Several lines of evidence further support the notion that production of capsule and HMV are separable. First, deletion of *rmpD* did not alter UA levels, suggesting that production of the capsular material is unaffected by this mutation. Second, strains that are hyper-HMV from overproduction of RmpD did not produce more UA than the WT strain. Third, *trans* expression of *rmpD* was able to restore HMV in the regulatory mutants (Δ*rmpA*, Δ*kvrA*, Δ*kvrB*, and Δ*rcsB*), that all have reduced *cps* expression and capsule production and are non-HMV. Each of these regulators activate transcription of the *rmpADC* promoter (28); thus, the loss of HMV in these mutants is most likely due to reduced expression of *rmpD*. Curiously, even though we can detect almost no expression from the *manC* promoter in the Δ*rcsB* strain (28), introduction of pRmpD in the Δ*rcsB* mutant, but not in the Δ*manC* mutant, results in hyper-HMV. Either very low levels of mannose-1-phosphate guanylyltransferase are sufficient for HMV production or HMV does not actually require this enzyme and the HMV defect in a Δ*manC* strain is an indirect effect of loss of this gene. Lastly, a recent study has identified several K. pneumoniae transposon mutants with normal capsule production and reduced HMV (42).

In mucoviscosity and adherence assays, the Δ*rmpD* strain behaves nearly identically to the capsule mutant Δ*manC*. Both mutants pellet tightly and are highly adherent to host cells. The hyper-HMV strains (WT and Δ*rmpD* with pRmpD) are essentially nonadherent, but the non-HMV Δ*manC* plus pRmpD strain remains highly adherent. This raises the question as to whether the antiadherence property of hvKp is dependent on capsule or on HMV. Given that the Δ*rmpD* strain is encapsulated, it appears that HMV is a more critical determinant for blocking adherence. This is consistent with the non-HMV Δ*rmpA* strain having a more severe virulence defect than the HMV-positive Δ*rmpC* strain in the mouse pneumonia model (28). Enumeration of bacteria internalized by J774A.1 cells showed a similar trend to adherence, except that the Δ*rmpD* strain was internalized less efficiently than the Δ*manC* strain. These data appear to suggest that HMV is a stronger deterrent to adherence than capsule, but that both capsule and HMV provide protection against phagocytosis in an additive manner. As this is the first attempt to examine strains with defects in HMV but not capsule production, similar analyses will need to be performed in other hvKp strains to determine if these trends are generally applicable or unique to KPPR1S. In the *in vivo* pneumonia model, the Δ*rmpD* mutant is severely attenuated, with a defect similar to that of the Δ*rmpA* mutant (28). This reinforces that HMV is an essential virulence factor in immunocompetent mice and suggests that capsule, while certainly providing some protection against host defenses, is insufficient in the absence of HMV for establishment of a lethal infection in mice. These data illustrate how HMV distinctly contributes to the hypervirulence of K. pneumoniae isolates. Support for this comes from reexamination of the virulence defects of the Δ*rmpA* and Δ*rmpC* strains. While it is possible that RmpA regulates additional virulence factors, the loss of *rmpD* expression in the Δ*rmpA* mutant likely contributes to the stronger virulence defect in the Δ*rmpA* mutant than in the Δ*rmpC* mutant. Similarly, analysis of KPPR1 genes essential for infection in a mouse pneumonia model identified mutations in VK055_5096 as deficient for virulence (33). This *orf* is located immediately upstream of *rmpA* (VK055_5097), and the transposon insertion quite likely impaired expression of the *rmp* locus. Furthermore, the virulence plasmid-borne *rmp* locus was found to be associated with liver abscess formation by NTUH-K2044 (43).

Complicating the notion that HMV is not simply a consequence of overabundant capsule production is that hyper-HMV did not occur in capsule-deficient mutants carrying pRmpD. This suggests that strains can be capsule positive/HMV positive or capsule positive/HMV negative but not capsule negative/HMV positive. One possible explanation for this is that the HMV material is capsular but that the export is altered in the presence of RmpD. This situation would mean that even reduced levels of biosynthetic enzymes such as those found in the regulatory mutants are sufficient to yield the extra polysaccharides. A second explanation is that HMV is a polysaccharide distinct from capsule, but that some *cps*-encoded functions are required to produce this material. A third possibility is that the HMV material is a modified form of capsule, and the presence of RmpD influences synthesis or export of the altered polysaccharide. That capsule-like material is part of HMV material is supported by the K2-positive staining of the HMV substance from a WT strain but not from non-HMV mutants (41).

To date, HMV has primarily been associated with hv K1 and K2 strains, but more than 130 capsule types of K. pneumoniae have been identified (44). Of significant concern is the number of recent reports of strains with both multidrug resistance and hv-associated genes, including *rmpA* (and quite likely *rmpD* and *rmpC*) (18–23). These strains are genetically quite distinct (including capsule type) from the hvKp that have been circulating, and it is not known to what degree acquisition of the *rmpADC* locus will impact HMV and virulence of these strains. While we have shown that RmpD from either a K2 or K1 strain can confer HMV in a K2 strain, it is not clear if there is capsule type specificity for this RmpD function. We also do not know, beyond a few *cps* genes, what, if any, other genes are necessary to confer HMV or if these genes are conserved in all K. pneumoniae strains. A better understanding of what is required for HMV and how genetic background influences the HMV-associated hypervirulent phenotypes will be important for determining the risks associated with carbapenem-resistant (CR) cKp strains that acquire *rmpADC*.

## MATERIALS AND METHODS

Additional experimental detail is available in Text S1 in the supplemental material.

### Bacterial strains, plasmids and growth conditions.

The strains and plasmids used in this work are listed in Table 1. Escherichia coli strains were grown in LB medium at 37°C. K. pneumoniae was grown at 37°C in M9 medium supplemented with 0.4% glucose and 0.2% Casamino Acids (M9-CAA). Unless otherwise noted, saturated overnight cultures were diluted to an OD_600_ of 0.2 and grown for 6 h. Antibiotics were used where appropriate: kanamycin (Kan), 50 μg/ml; rifampin (Rif), 30 μg/ml; spectinomycin (Sp), 50 μg/ml. For expression of genes cloned into pMWO-078, 100 ng/ml anhydrous tetracycline (aTc) was added to the medium at the time of subculture. The primers used for cloning are listed in Table S1. In-frame gene deletions in K. pneumoniae were constructed by allelic exchange using pKAS46-based plasmids as described previously (28). Complementation plasmids were constructed using pMWO-078 (45). Plasmids containing promoter-*gfp* fusions were cloned in pPROBE-tagless (46). The *gfp* reporter and complementation plasmids were introduced into K. pneumoniae by electroporation as described previously (28).

**TABLE 1:** Strains and plasmids used in this work

Strain or plasmid	Relevant genotype	Reference or source
Strains		
E. coli		
DH5α	F^−^ p80Δ*lacZ*M15 Δ(*lacZYA-argF*)*U169 deoP recA1 endA1 hsdR17* (r_K_^−^ m_K_^−^)	Invitrogen
S17-1λpir	Tp^r^ Str^r^ *recA thi pro hsdR hsdM*^+^ RP4::2-Tc::Mu::Km Tn*7* λpir lysogen	47
K. pneumoniae		
KPPR1S	ATCC 43816, Rif^r^, Str^r^	48
VK487	KPPR1S, Δ*rmpC*	28
VK506	KPPR1S, Δ*manC*	28
VK429	KPPR1S, Δ*rmpADC*	This work
VK646	KPPR1S, Δ*wcaJ*	This work
VK637	KPPR1S, Δ*rmpD*	This work
VK680	VK637, *rmpD* restored at native site	This work
VK248	KPPR1S, Δ*rcsB*	28
VK277	KPPR1S, Δ*kvrA*	36
VK410	KPPR1S, Δ*kvrB*	36
VK093	KPPR1, Str^s^, Kan^r^, *wecA*::Tn*5*	38
Plasmids		
pPROBE	Kan^r^; *gfp* transcriptional reporter vector	46
pKAS46	Kan^r^; MobRP4 *ori*R6K, cloning vector	49
pJH026	pPROBE with constitutive *em7* promoter	50
pMWO-078	Sp^r^; p15A *ori* cloning vector, *tetO*	45
pCB109	*rmpADC* in frame deletion in pKAS46	28
pPROBE-*manC*	*manC* promoter region in pPROBE	36
pPROBE-*galF*	*galF* promoter region in pPROBE	36
pPROBE-*wzi*	*wzi* promoter region in pPROBE	36
pKW184/pRmpA	*rmpA* in pMWO-078	28
pKW185/pRmpC	*rmpC* in pMWO-078	28
pKW186/pRmpADC	*rmpADC* in pMWO-078	28
pKW173/pRcsB	*rcsB* in pMWO-078	28
pTM006/pKvrA	*kvrA* in pMWO-078	28
pTM007/pKvrB	*kvrB* in pMWO-078	28
pLPT008	*rmpD* deletion in pKAS46	This work
pLPT007/pRmpD	*rmpD* in pMWO-078	This work
pLPT006/pORF5098	VK055_5098 in pMWO-078	This work
pLPT017	*wcaJ* deletion in pKAS46	This work
pKW190	*rmpD*-2×FLAG in pMWO-078	This work
pKW198	*rmpD* from Kp52145 in pMWO-078	This work
pKW199	*rmpD* from NTUH-K2044 plasmid in pMWO-078	This work
pKW200	*rmpD* from NTUH-K2044 chromosome in pMWO-078	This work

10.1128/mBio.01750-20.6 TABLE S1 Primers and synthetic genes used in this work. Download Table S1, DOCX file, 0.01 MB. Copyright © 2020 Walker et al. 2020 Walker et al. This content is distributed under the terms of the Creative Commons Attribution 4.0 International license.

### Transcriptional *gfp* reporter assays.

Relative fluorescent units (RFU) and OD_600_ were measured from bacterial cultures diluted 1:10 by using a Synergy H1 plate reader (Bio-Tek, Winooski, WI) and a Bio-Rad spectrophotometer (Bio-Rad, Hercules, CA), respectively. Data are presented as RFU/OD_600_, normalized to the activity from the wild-type strain in each assay.

### Assessment of capsule production and HMV.

Uronic acid was measured essentially as described previously (38). Mucoviscosity of liquid cultures was determined by measuring the OD_600_ of the culture supernatant following low-speed centrifugation as described previously (28). Centrifugation was chosen over the string test because it is semiquantitative, whereas the string test is purely qualitative. All strains that pellet tightly are string test negative, while those with turbid supernatants are string test positive.

### Immunoblotting.

Whole-cell lysates from cultures grown in M9-CAA with aTc for 6 h were separated on 15% SDS-PAGE gels, transferred to polyvinylidene difluoride (PVDF) membranes, probed with α-FLAG antibody (Sigma, M2 monoclonal antibody), and detected with chemiluminescence.

### Adherence and internalization assays.

Adherence assays were performed essentially as described previously (36) using J774A.1 cells. For adherence, the cells were pretreated with cytochalasin D 1 h prior to inoculation to prevent internalization of the bacteria. For internalization, cytochalasin D was omitted, and the wells were treated with 200 μg/ml gentamicin after a 1-h coincubation. Recovered bacteria (as CFU) are reported as a percentage of the inoculum CFU.

### Murine pneumonia model.

All animal studies were approved by the Institutional Care and Use of Committee of UNC-CH. Anesthetized C57BL/6J mice (Jackson Laboratories, Bar Harbor, ME) were inoculated with 2 × 10^4^ CFU intranasally as described previously (36). Mice were sacrificed at 24 h and 72 h postinoculation; lungs and spleens removed for determination of bacterial enumeration. The data are presented as CFU/gram tissue.

### India ink staining.

Bacterial cultures carrying a constitutively expressing *gfp* reporter (pJH026) were grown as for all other assays. Equal volumes of culture and India ink were mixed on a glass slide and overlaid with a coverslip. Microscopy was performed using a Keyence BZ-X810 microscope at ×1,000 magnification.

### Statistics and replicates.

Statistical tests for each experiment are given in the figure legends and were performed using GraphPad Prism 8.2. For every assay, a minimum of three assays were performed, each with biological replicates. Typically, a representative experiment is presented. In all bar graphs, bars are average values and error bars indicate standard deviations (*n* ≥ 3).
