# Supplementary material for: The Small Protein RmpD Drives Hypermucoviscosity in Klebsiella pneumoniae
Source: mBio. 2020 Sep 22;11(5):e01750-20. doi: 10.1128/mBio.01750-20 (PMC7512549; doi:10.1128/mBio.01750-20)
Supplement: FIG S1 [file mBio.01750-20-sf001.pdf]

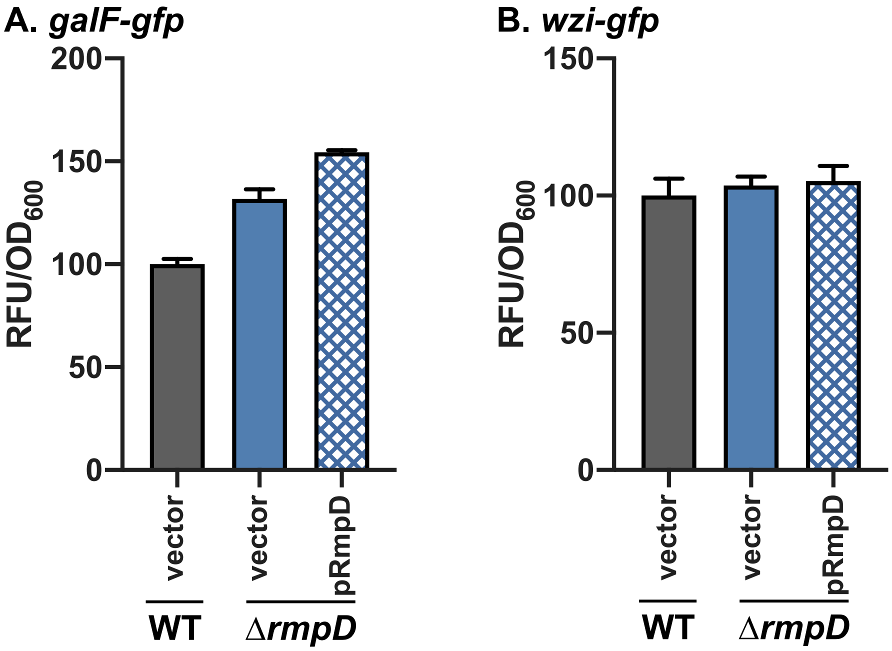

Figure S1. Expression of other known capsule promoters is not deficient in the Δ*rmpD* strain. Promoter regions upstream of *galF* (A) and *wzi* (B) were cloned into pPROBE and fluorescense was measured as an indicator of promoter activity. Values were normalized to WT for each promoter. Neither promoter is decreased in the Δ*rmpD* strain, and over expression of pRmpD does not change expression compared to Δ*rmpD* with the vector control. Data were obtained as for Figures 1 and 2. The increase in expression of *galF* in the Δ*rmpD* mutant is significantly higher than WT ($p<0.0001$).
