# Supplementary material for: The Small Protein RmpD Drives Hypermucoviscosity in Klebsiella pneumoniae
Source: mBio. 2020 Sep 22;11(5):e01750-20. doi: 10.1128/mBio.01750-20 (PMC7512549; doi:10.1128/mBio.01750-20)
Supplement: FIG S2 [file mBio.01750-20-sf002.pdf]

A. WT + vector **B. WT + pRmpD**

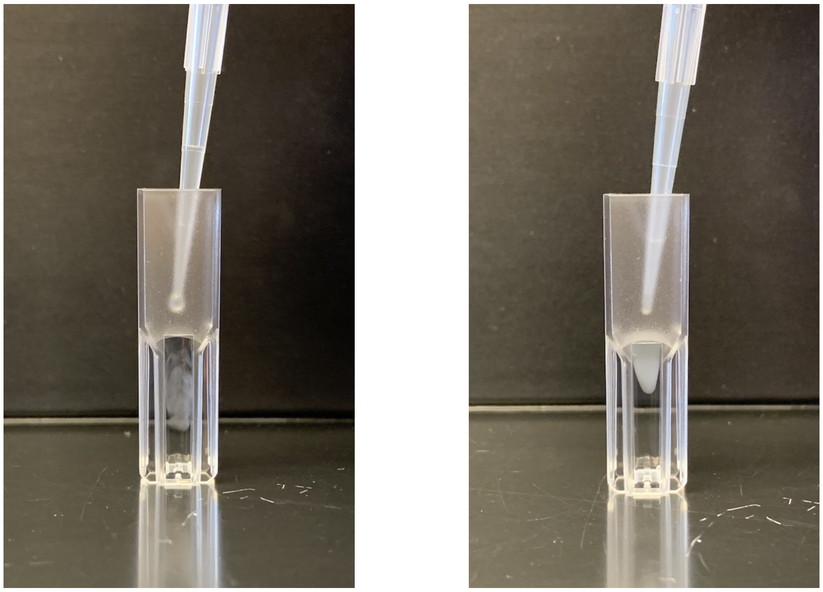

Figure S2. *K. pneumoniae* overexpressing *rmpD* are thick and syrupy. Cultures were grown with induction by aTc as described in Materials & Methods. 100 µl of culture was dropped into 900 µl 1xPBS in a spectrophotometer cuvette.
