# Supplementary material for: The Small Protein RmpD Drives Hypermucoviscosity in Klebsiella pneumoniae
Source: mBio. 2020 Sep 22;11(5):e01750-20. doi: 10.1128/mBio.01750-20 (PMC7512549; doi:10.1128/mBio.01750-20)
Supplement: FIG S3 [file mBio.01750-20-sf003.pdf]

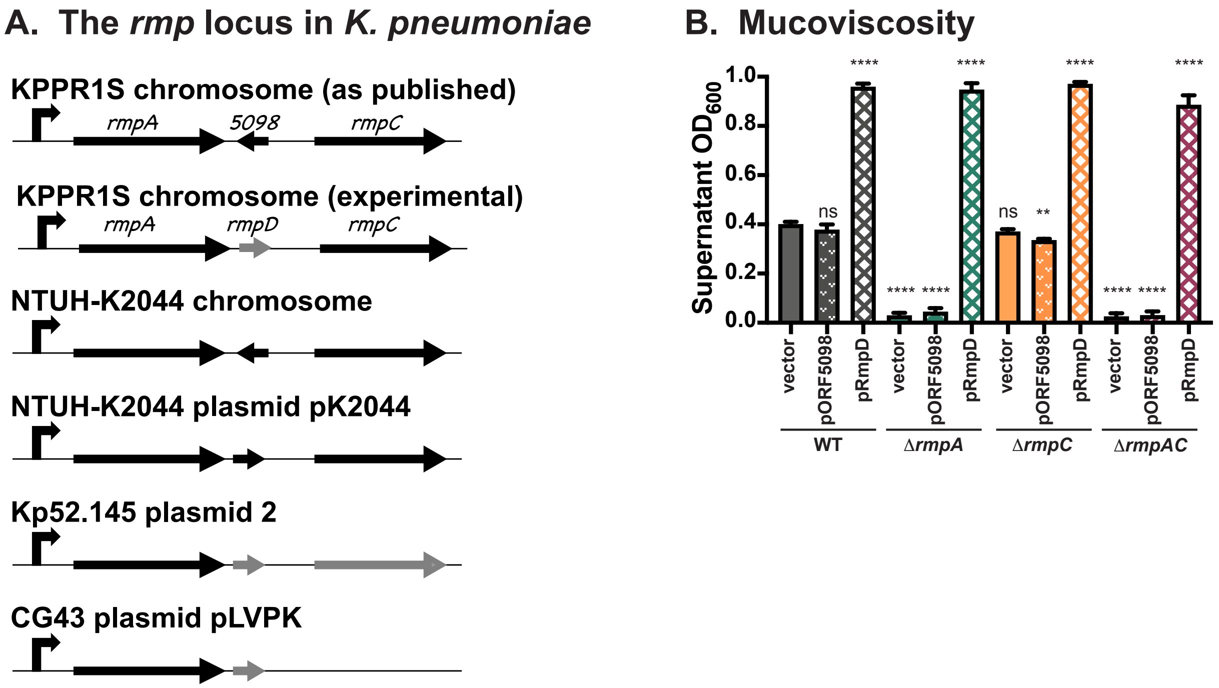

Figure S3. Organization of the *rmp* locus in *K. pneumoniae*. (A) Schematic of *rmp* loci from several *hv*Kp strains. Black genes, annotated ORFs in NCBI; gray genes, annotated ORFs in Geneious Prime 2019 software. Accession numbers for these genomes are in Supplemental Methods. (B) The ORF encoded on the opposite strand (VK055_5098) with putative RBS was cloned into pMWO-078 (pORF5098), transformed into the indicated strains and tested for effects on mucoviscosity. It had no impact in any strain, indicating that this ORF does not contribute to the HMV phenotype.
