# Supplementary material for: The Small Protein RmpD Drives Hypermucoviscosity in Klebsiella pneumoniae
Source: mBio. 2020 Sep 22;11(5):e01750-20. doi: 10.1128/mBio.01750-20 (PMC7512549; doi:10.1128/mBio.01750-20)
Supplement: TEXT S1 [file mBio.01750-20-s0001.docx]

**Supplemental Methods for:**

**The small protein RmpD drives hypermucoviscosity in *Klebsiella pneumoniae***

Kimberly A. Walker ^a1^, Logan P. Treat ^a^, Victoria E. Sepúlveda^a^, and Virginia L. Miller ^a,b^

Departments of Microbiology and Immunology ^a^ and Genetics^b^

University of North Carolina School of Medicine, Chapel Hill, NC 27599

^1^ Corresponding author: kawalker@med.unc.edu

**DETAILED MATERIALS & METHODS**

**Bacterial strains and growth conditions.** The strains and plasmids used in this work are listed in Table S1. *E. coli* strains were grown in LB medium (10g/L tryptone, 5g/L yeast extract, 10g/L sodium chloride) at 37°C. *K. pneumoniae* were grown at 37°C in M9 medium (1) supplemented with 0.4% glucose and 0.2% casamino acids (M9-CAA). The following antibiotics were used where appropriate: kanamycin (Kan), 50 µg/ml; rifampicin (Rif), 30 µg/ml, spectinomycin (Sp), 50 µg/ml, streptomycin (Strep), 2500 µg/ml. For expression of genes cloned into pMWO-078, 100 ng/ml anhydrous tetracycline (aTc) was added to the media at the time of subculture.

**Plasmid and strain construction.** The primers used for each construct are listed in Table S2. All plasmids were confirmed by sequencing. In-frame and clean gene deletions in *K. pneumoniae* were constructed by allelic exchange using pKAS46-based plasmids as described (2). Briefly, 500 bp flanking regions were amplified, digested and cloned into pKAS46 digested with EcoRV and NotI. The resulting plasmids were introduced into *K. pneumoniae* by conjugation, selected on LB agar with Rif_30_/Kan_50_. Counterselection for double recombinants was carried out on LB agar with Strep_2500_. Strep^R^/Kan^S^ colonies were screened for the manipulated gene by PCR. Complementation plasmids were constructed using pMWO-078 (3), which contains an aTc-inducible promoter; the vector was digested with SalI and BamHI. Some clones were generated by Gibson Assembly (New England Biolabs), and the amplicons were not digested prior to cloning. Plasmids containing various promoter-*gfp* fusions were cloned in pPROBE-tagless (4). The *gfp* reporter and complementation plasmids were introduced into *K. pneumoniae* by electroporation as described (2).

**Transcriptional *gfp* reporter assays.** Saturated cultures grown overnight (~16 hours) in M9-CAA were subcultured into fresh medium to an initial OD_600_ of 0.2 and grown for 6 h. Relative fluorescent units (RFU) and OD_600_ were measured from bacterial cultures diluted 1:10 using a Synergy H1 plate reader (Bio-Tek, Winooski, WI) and a Bio-Rad spectrophotometer (Bio-Rad, Hercules, CA), respectively. Data are presented as RFU/OD_600_, normalized to the activity from the wild type strain in each assay.

**Assessment of capsule production and HMV.** Saturated cultures grown overnight in M9-CAA were subcultured into fresh medium to an initial OD_600_ of 0.2 and grown for 6 h. Uronic acid was measured essentially as described (2) using standard protocols (5, 6); polysaccharides were allowed to precipitate overnight at 4°C. Mucoviscosity of the liquid cultures was determined by measuring the OD_600_ of the culture supernatant following low-speed centrifugation as described (2). After 6h subculture, samples were diluted to ~1 OD_600_/ml (2 ml volume) in 1xPBS and centrifuged at 1000 x *g* for 5 minutes. An aliquot was removed prior to centrifugation to measure the pre-spin OD_600_, and OD_600_ was measured after the spin. The data are presented as Post-spin OD_600_/Pre-spin OD_600_.

**Immunoblotting.** Saturated cultures grown overnight in M9-CAA were subcultured into fresh medium to an initial OD_600_ of 0.2 and grown for 6 h. Whole cell lysates were separated on 15% SDS-PAGE gels and transferred to PVDF membranes. Membranes were blocked overnight in PBST/5% milk, then incubated for 1 h in PBST/5% milk with α-FLAG antibody (1:10,000; Sigma; M2 monoclonal). After rinsing, the membrane was incubated for 1 h in PBST/5% milk with goat α-mouse secondary antibody (1:50,000; Sigma), then rinsed, and detected with Clarity ECL detection reagents (Bio-Rad).

**Adherence assays.** J774A.1 cells cultured in DMEM with 10% FBS, sodium pyruvate and NEAA were seeded into 24-well plates at a density of 5x10^5^ cells per well and incubated overnight at 37°C, 5% CO_2_. Saturated cultures grown overnight in M9-CAA were subcultured into fresh medium with aTc to an initial OD_600_ of 0.2 and grown for 6 h. One hour prior to the assays, the media was replaced with fresh DMEM medium containing 2 µM cytochalasin D (Sigma, St. Louis, MO) to inhibit internalization of bacteria. Cells were inoculated with the desired *K. pneumoniae* strains at an MOI of 50 (~2.5x10^7^ CFU). After one hour, the wells were gently rinsed three times with sterile 1xPBS and then lysed with 200µl 0.5% saponin in PBS for 5 minutes. 800 µl 1XPBS was added to each well for a final volume of 1 ml, then the lysate was diluted and plated on LB agar to determine the number of CFU. Adherent bacteria (recovered CFU) are presented as a percent of the inoculum. Each assay was performed in quadruplicate, and each strain was tested in at least 3 independent assays with WT and ∆*manC* as controls in each assay.

**Internalization assays.** J774A.1 cells were cultured and seeded and inoculated as for adherence assays, but cytochalasin D was not added. After one hour of co-incubation with bacteria, the wells were washed three times with 1xPBS, then fresh DMEM containing 200 µg/ml gentamicin was added. After 30 minutes, the media was removed; cells were lysed, diluted, plated and calculated as for adherence assays.

**Murine pneumonia model.** All animal studies were approved by the Institutional Care and Use of Committee of UNC-CH (protocol 17-033). Mice had unlimited access to food and water. Inoculated mice were monitored daily, and mice were euthanized upon showing signs of morbidity. Five-to-eight-week old female C57BL/6j mice (Jackson Laboratories, Bar Harbor, ME) were anesthetized with ketamine/xylazine by intraperitoneal (i.p.) injection and inoculated with a 20 µl suspension of 2 x 10^4^ CFU in 1xPBS intranasally as described (7). Mice were euthanized by i.p. injection with sodium pentobarbital at 24h and 72h post-inoculation; lungs and spleens removed for determination of bacterial enumeration. Organ weights were recorded and the data presented as CFU/gram tissue.

**Sequence alignments.** Sequences for various *K. pneumoniae* strains were retrieved from GenBank. The DNA sequence for *rmpD* was located by looking at ORFs assigned by Geneious Prime 2019 downstream of *rmpA*. These sequences were then translated and aligned with Geneious Prime. Accession numbers are as follows: KPPR1S, CP009208.1 (8); NTUH-c, AP006725(9); NTUH-p, AP006726 (9); CG43, AY378100.1 (pLVPK) (10); Kp52.145, NZ_FO834905 (plasmid II) (11).

**RT-PCR.** The RNA used to generate the cDNA was isolated from KPPR1S grown in LB from a previously published data set (7). cDNA synthesis was generated from 1 µg RNA using the iScript cDNA synthesis kit (Bio-Rad). Qualitative RT-PCR was performed using ~25ng cDNA, 500 nM primers and SsoAdvance SYBR green Supermix (Bio-Rad). Primers pairs were as follows: rmpA-C, CB472/CB498, rmpA-D, CB472/rmpD-delB, rmpD-C, MP355/CB498 (sequences in Table S2). The products were separated on 1% agarose gels and stained with ethidium bromide.

**REFERENCES**

1. Sambrook J, Fritsch EF, Maniatis T (1989) *Molecular cloning* (Cold Spring Harbor Laboratory Press).

2. Walker KA, et al. (2019) A *Klebsiella pneumoniae* Regulatory Mutant Has Reduced Capsule Expression but Retains Hypermucoviscosity. *MBio* 10(2):e00089–19.

3. Obrist MW, Miller VL (2012) Low copy expression vectors for use in *Yersinia* sp. and related organisms. *Plasmid* 68:33–42.

4. Miller WG, Leveau JH, Lindow SE (2000) Improved *gfp* and *inaZ* broad-host-range promoter-probe vectors. *Mol Plant Microbe Interact* 13(11):1243–1250.

5. Blumenkrantz N, Asboe-Hansen G (1973) New method for quantitative determination of uronic acids. *Anal Biochem* 54(2):484–489.

6. Domenico P, Schwartz S, Cunha BA (1989) Reduction of capsular polysaccharide production in *Klebsiella pneumoniae* by sodium salicylate. *Infect Immun* 57(12):3778–3782.

7. Palacios M, et al. (2018) Identification of Two Regulators of Virulence That Are Conserved in *Klebsiella pneumoniae* Classical and Hypervirulent Strains. *MBio* 9(4):e01443–18.

8. Broberg CA, Wu W, Cavalcoli JD, Miller VL, Bachman MA (2014) Complete Genome Sequence of *Klebsiella pneumoniae* Strain ATCC 43816 KPPR1, a Rifampin-Resistant Mutant Commonly Used in Animal, Genetic, and Molecular Biology Studies. *Genome Announc* 2(5):e00924–14.

9. Wu K-M, et al. (2009) Genome sequencing and comparative analysis of *Klebsiella pneumoniae* NTUH-K2044, a strain causing liver abscess and meningitis. *J Bacteriol* 191(14):4492–4501.

10. Chen Y-T, et al. (2004) Sequencing and analysis of the large virulence plasmid pLVPK of *Klebsiella pneumoniae* CG43. *Gene* 337:189–198.

11. Lery LMS, et al. (2014) Comparative analysis of *Klebsiella pneumoniae* genomes identifies a phospholipase D family protein as a novel virulence factor. *BMC Biol* 12:41.

12. Miller VL, Mekalanos JJ (1988) A novel suicide vector and its use in construction of insertion mutations: osmoregulation of outer membrane proteins and virulence determinants in *Vibrio cholerae* requires *toxR*. *J Bacteriol* 170(6):2575–2583.

13. Palacios M, Broberg CA, Walker KA, Miller VL (2017) A Serendipitous Mutation Reveals the Severe Virulence Defect of a *Klebsiella pneumoniae fepB* Mutant. *mSphere* 2(4):e00341–17.

14. Lawlor MS, Hsu J, Rick PD, Miller VL (2005) Identification of *Klebsiella pneumoniae* virulence determinants using an intranasal infection model. *Mol Microbiol* 58(4):1054–1073.

15. Skorupski K, Taylor RK (1996) Positive selection vectors for allelic exchange. *Gene* 169(1):47–52.

16. Walker KA, Maltez VI, Hall JD, Vitko NP, Miller VL (2013) A Phenotype at Last: Essential Role for the *Yersinia enterocolitica* Ysa Type III Secretion System in a *Drosophila melanogaster* S2 Cell Model. *Infect Immun* 81(7):2478–2487.
