# Supplementary material for: The Small Protein RmpD Drives Hypermucoviscosity in Klebsiella pneumoniae
Source: mBio. 2020 Sep 22;11(5):e01750-20. doi: 10.1128/mBio.01750-20 (PMC7512549; doi:10.1128/mBio.01750-20)
Supplement: FIG S4 [file mBio.01750-20-sf004.pdf]

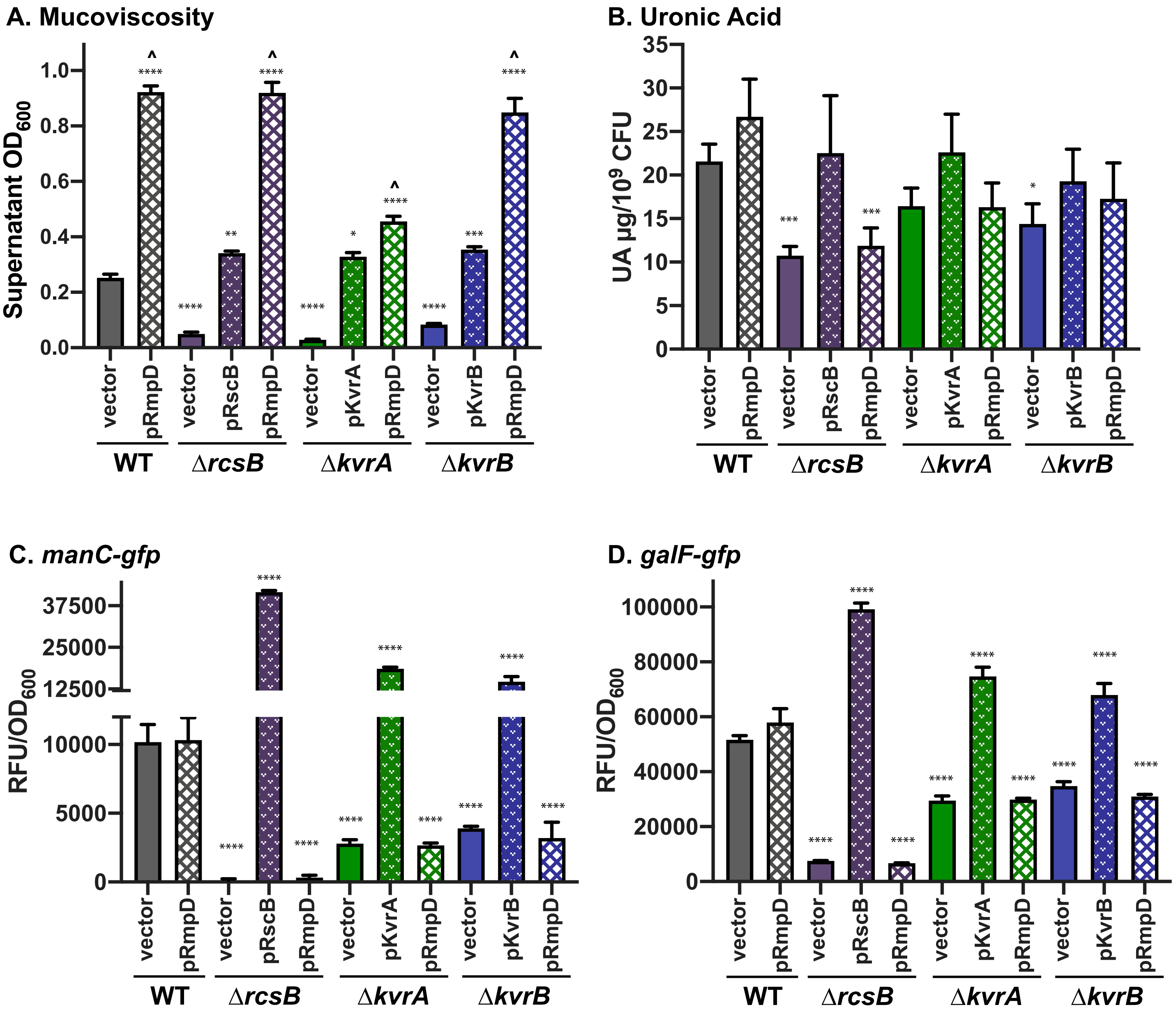

Figure S4. Plasmids pKvrA, pKvrB, pRcsB functionally complement their respective mutants. Mucoviscosity (A), uronic acid (B) *manC* (C) and *galF* (D) expression were measured as described in Materials and Methods except expression data is not normalized to WT.
