# Supplementary material for: The Small Protein RmpD Drives Hypermucoviscosity in Klebsiella pneumoniae
Source: mBio. 2020 Sep 22;11(5):e01750-20. doi: 10.1128/mBio.01750-20 (PMC7512549; doi:10.1128/mBio.01750-20)
Supplement: FIG S5 [file mBio.01750-20-sf005.pdf]

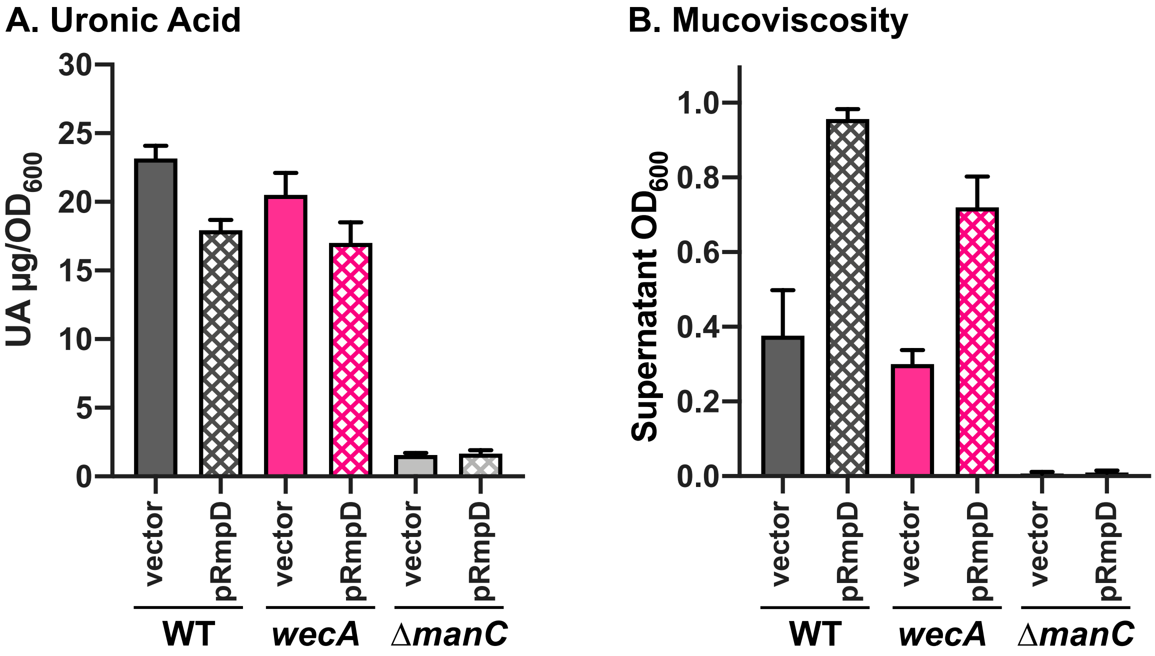

Figure S5. LPS and ECA are not necessary for HMV. Uronic acid (A) and mucoviscosity (B) were measured as in Figure 3. This data was collected preliminarily, using OD to normalize the UA concentrations. We typically normalize to CFU, but decided not to add that until we had an idea of the results. Due to COVID-19, our lab closed before we could repeat with CFU data included. The above assays were performed in triplicate, with three biological replicates in each assay, so we are confident in the interpretations of this data described in the Results and Discussion and did not think that statistics were needed to strengthen the analysis.
