# Supplementary material for: The Small Protein RmpD Drives Hypermucoviscosity in Klebsiella pneumoniae
Source: mBio. 2020 Sep 22;11(5):e01750-20. doi: 10.1128/mBio.01750-20 (PMC7512549; doi:10.1128/mBio.01750-20)
Supplement: TABLE S1 [file mBio.01750-20-st001.docx]

**The small protein RmpD drives hypermucoviscosity in *Klebsiella pneumoniae***

Kimberly A. Walker, Logan P. Treat, Victoria E. Sepúlveda, and Virginia L. Miller

**Table S1. Primers and synthetic genes used in this work**

**Name Sequence^a^(5’→3’) Use^b^**

LT016 GATCTGCGCGCGATCGATATCCGCGGTGAAATGCATGTGTC F pLPT017 5’ flank

LT017 GGAGCATCTAATCAATAAGCAGACTGAGATATTGTCATAATTCAATC R pLPT017 5’ flank

LT018 TCTGCTTATTGATTAGATGCTCCTTAAGACAAGGTTTGTGC F pLPT017 3’ flank

LT019 GCGCCAGCTGCAGGCGGCCGCGCAGAGAGTTCACGGTTACG R pLPT017 3’ flank

rmpD-delA GATCTGCGCGCGATCGATATCCTAAAGCAGTTAACTGGACTACC F pLPT008 5’ flank

rmpD-delB ATAATGTATAGTGACCCTATAATAAATAAATGAAAGAGTGC R pLPT008 5’ flank; RT-PCR

rmpD-delC ATAGGGTCACTATACATTATAATGTATCTCCAGCAAATGAG F pLPT008 3’ flank

rmpD-delD CGCCAGCTGCAGGCGGCCGCCCGGTCTTATTAAGAATATTCC R pLPT008 3’ flank

KW415 TTCCTGCAGCCCGGGGGATCCGGTTGATGAAAGATGTCTCATG F pLPT007

KW416 GCTCCACCGCGGTGGCGGCCGCGCTGATATCATTTATTGAATGTG R pLPT007

KW417 TTCCTGCAGCCCGGGGGATCCGCTGATATCATTTATTGAATGTG F pLPT006

KW418 GCTCCACCGCGGTGGCGGCCGCGTGAAAGCACTCTTTCATTTAT R pLPT006

KW419 GCTCTAGATGATGTTAATTCCGATGAGGGTGG F pKW196

KW420 CGGAATTCGCACAGAACATTGATAAATAG R pKW196

CB472 GATCATCTGCAGGGTTGATGAAAGATGTCTCATGF RT-PCR

CB498 GATCATGTCGACCACAATCTCATTATTTTTCTTACC RT-PCR

MP355 TGCATATCTAGAGATATTCCGCTCGCACAGAACAT RT-PCR

**Synthetic genes**

rmpD-2xFLAG for pKW190 TTCCTGCAGCCCGGGGGATCCGGTTGATGAAAGATGTCTCATGCTAGGTATTTAGAAAAAAAGGGGAGGAGGGGGTGAAAGCACTCTTTCATTTATTTATTTTTTTATTTCTGTTTTATATATCAGTTTATTGTTTTTATTCATATGTGTCAGATAGAAGAAGAATAAAAAAAATATTCCGCTCGCACAGAACATTGATAAATAGACGAAAAAAATCAAACTTAATAAAATATATCACATTCAATAAAGACTACAAGGACGACGATGACAAGGACTATAAAGATGATGACGATAAATGAGCGGCCGCCACCGCGGTGGAGC

52145 rmpD for pKW199 TTCCTGCAGCCCGGGGGATCCGGTTGATGAAAGATGGCTCATGCCAAGTATTTAGGTAAAAAAAAGGGGGAGGGGATGT

GAAGGAACTCTATTATGTATTCATTGTTTTCTTTTTGTTTTTTTTGTCAGTTTATAATTTTTATTCTTATACTTCAGATAGAATAAAAAGAAAAAGAATATTCCGCTCGCGTAGAAAATCGATGAATAGACGCAAAAAATTCAAACTATTATAGGCGGCCGCCACCGCGGTGGAGC

NTUHp rmpD for pKW198 TTCCTGCAGCCCGGGGGATCCGGTTGATGAAAGATGGCTCATGCCAAGTATTTAGGTAAAAAAGGGGAGGGGATGTGAA

GGATCTCTTTTATTTATTTATTGCTTTTTTTTTGTTTTTTTTGTCAGTTTATAATTCATATTCTTATATTTCAGACAGAAGAAAAATAAAAAGAATCTTCCGCTCGCGCAGAAAAGCGATGAATAGACGCAAAAAAAAATTCAAACTATTATAGGCGGCCGCCACCGCGGTGGAGC

NTUHc rmpD for pKW200 TTCCTGCAGCCCGGGGGATCCGGTTGATGAAAGATGTCTCATGCCAGGTATTTAGAAAAAAAGGGGAGGAGGGGGTGAAAGCACTCTTTCATTTATTTATTGTTTTATTTCTGTTTTATATATCAGTTTATTATTTTTATTCATATGTGTCAGATAGAAGAAGAATAAAAAAATTATTCCGCTCGCACAGAACATTGATAAATAGACGAAAAAATCAAACTTAAGCGGCCGCCACCGCGGTGGAGC

_____________________________________________________________________________________________

^a^ Blue nt are overhangs for Gibson cloning. Underlined nt indicate restriction sites.

^b^ F, forward primer; R, reverse primer
